# An Improved Genome Assembly of *Azadirachta indica* A. Juss.

**DOI:** 10.1534/g3.116.030056

**Published:** 2016-04-18

**Authors:** Neeraja M. Krishnan, Prachi Jain, Saurabh Gupta, Arun K. Hariharan, Binay Panda

**Affiliations:** * Ganit Labs, Bio-IT Centre, Institute of Bioinformatics and Applied Biotechnology, Bangalore 560100, India; † Strand Life Sciences, Bangalore 560024, India

**Keywords:** PASA, mate-pair, assembly, *FDFT1*, *SQLE*

## Abstract

Neem (*Azadirachta indica* A. Juss.), an evergreen tree of the Meliaceae family, is known for its medicinal, cosmetic, pesticidal and insecticidal properties. We had previously sequenced and published the draft genome of a neem plant, using mainly short read sequencing data. In this report, we present an improved genome assembly generated using additional short reads from Illumina and long reads from Pacific Biosciences SMRT sequencer. We assembled short reads and error-corrected long reads using Platanus, an assembler designed to perform well for heterozygous genomes. The updated genome assembly (v2.0) yielded 3- and 3.5-fold increase in N50 and N75, respectively; 2.6-fold decrease in the total number of scaffolds; 1.25-fold increase in the number of valid transcriptome alignments; 13.4-fold less misassembly and 1.85-fold increase in the percentage repeat, over the earlier assembly (v1.0). The current assembly also maps better to the genes known to be involved in the terpenoid biosynthesis pathway. Together, the data represent an improved assembly of the *A. indica* genome.

High-throughput sequencing platforms, especially those based on short read technology, have enabled sequencing of many plant genomes (Michael and Jackson 2013). This has substantially improved our understanding of genome organization, evolution and complexity in different plant species. However, most first generation genome assemblies are draft and incomplete assemblies. The correctness and accuracy of genome assembly depends on the length of the sequencing reads, errors generated during sequencing, and the accuracy of the computational tools (assemblers and downstream annotation pipelines) used. Additionally, most genome assemblers are not suitable to assemble genomes of heterozygous plants, a characteristic feature of most plants in the wild (Kajitani *et al.* 2014). Draft assemblies often bear significant gaps and errors, yielding less accurate gene predictions and annotations. This is compounded by the usage of incomplete training sets with gene prediction algorithms and absence of a representative transcriptome that can correctly anchor to the genome. Therefore, it is imperative to improve the quality of draft genome assemblies with the help of longer reads using genome assemblers tailored to handle heterozygosity, and make gene predictions using updated training sets and gene annotations using combinatorial approaches not fully reliant on sequence similarity such as Basic Local Alignment Search Tool (BLAST).

Neem (*Azadirachta indica* A. Juss.), belonging to the order Rutales, family Meliaceae, is an important woody angiosperm, given its many medicinal and agrochemical uses. We had previously sequenced and reported the draft genome and five organ-specific transcriptomes (Krishnan *et al.* 2011, 2012) of a neem tree. The neem genome was the 38th plant genome to be sequenced (Michael and Jackson 2013). The genome assembly was generated using short paired-end reads (76 bases or shorter) from Illumina GAIIx with a first generation genome assembler, SOAPdenovo (Li *et al.* 2010). This was followed by genome annotation and gene prediction analysis, analysis of repeat elements, phylogenetic analysis, and gene expression studies (Krishnan *et al.* 2012). In the current report, we have improved the quality of the neem genome assembly by (a) using additional long-insert libraries from Illumina Hiseq, (b) using long reads from a third generation sequencer by Pacific Biosciences (PacBio), (c) using LoRDEC (Salmela and Rivals 2014), an algorithm that takes short reads from Illumina and uses those to correct errors in the PacBio reads, and (d) assembling the genome with short and error-corrected long reads using Platanus (Kajitani *et al.* 2014), which is better suited to assemble heterozygous genomes. We reassembled all five organ-specific RNA libraries into a pooled representative transcriptome, using Trinity (Grabherr *et al.* 2011; Haas *et al.* 2013), and employed the Program to Assemble Spliced Alignments (PASA, Haas *et al.* 2003) to benchmark the completeness of previous (v1.0), intermediate, and current (v2.0) genome assemblies based on their mappability to this transcriptome. We also performed gene prediction analyses with GlimmerHMM (Majoros *et al.* 2004, v3.0.4) using updated training sets from *Citrus* species, which were found to be evolutionarily closer to neem by our earlier phylogenetic analyses (Krishnan *et al.* 2012). Building on our draft assembly, here, we present data on different assembly parameters, accuracy, gaps, gene predictions, and the total repeat content as evidence toward an improved neem genome assembly.

## Materials and Methods

### Assembly

In addition to the Illumina read libraries used for assembling the previously published draft neem genome (Krishnan *et al.* 2012), four more libraries were used for updating the assembly. We included reads from three Illumina mate-pair (with insert sizes 4 kb, 6 kb, and 10 kb) and one PacBio (average read length > 2kb, varying up to 17.64 kb) libraries. Details of all libraries used are presented in Supplemental Material, Table S1.

We preprocessed all the libraries as follows. In the case of Illumina libraries, exact read duplicates were removed using the ’in silico normalization’ utility from Trinity. For PacBio, reads were error-corrected using LoRDEC v0.4.1 based on the two paired-end Illumina libraries (Table S1). kmers ranging from 19 to 36 were tested for error-correction. We made an effort to assemble intact PacBio reads following error-correction using the PacBioToCA (Koren *et al.* 2012) pipeline and Celera WGS assembler v7.1 (Myers *et al.* 2000). However, this process was CPU- and RAM-intensive, and also resulted in a suboptimal assembly (data not shown). We, therefore, converted the PacBio reads, with and without error-correction, into Illumina-like paired-end reads (read lengths of 100 bases and average insert size of 350 bases) using SInC’s read generator (Pattnaik *et al.* 2014), which could be easily assembled using SOAPdenovo, SOAPdenovo2 (Luo *et al.* 2012), and Platanus. Converting PacBio reads to Illumina-like reads did not nullify the advantage of the long reads, in terms of contiguity (File S1).

We produced 13 intermediate assemblies (Table S2) for quality comparison, as follows: (a) reassembly of the published version using SOAPdenovo with Illumina short reads (R.S1/v1.0); (b) assembly using additional Illumina libraries using SOAPdenovo2 (S2.DUP); (c) assembly of all Illumina duplicate-removed libraries using SOAPdenovo2 (S2); (d) assembly, using SOAPdenovo2, of all Illumina duplicate-removed libraries along with the error-corrected PacBio reads (S2.ecPB.21 and S2.ecPB.32, using kmers 21 and 32, respectively); (e) assembly using Platanus of all Illumina duplicate-removed libraries alone (P), or along with either the error-corrected PacBio reads using 19- (P.ecPB.19), 21- (P.ecPB.21), 32- (P.ecPB.32), and 36-mers (P.ecPB.36), or along with uncorrected PacBio reads (P.ucPB) f) assembly and gap-closing, using Platanus, of all Illumina duplicate-removed libraries and the PacBio library with (P.ecPB.32.gc/v2.0; kmer = 32) or without (P.ucPB.gc) error-correction.

All assembly QCs were performed using QUAST v2.3 (Gurevich *et al.* 2013). The assembly NG50 was estimated assuming the neem genome size to be 364 Mb (Krishnan *et al.* 2012). We refer to the R.S1 assembly as v1.0 (previous) and the P.ecPB.32.gc assembly as the improved v2.0 (current) in our comparisons statistics below.

### Assembly mapping to transcriptome using PASA

PASA r20140417 was used to compare and evaluate all the assemblies. The representative neem transcriptome was assembled *de novo* using Trinity v2.0.6 with five tissue-specific published RNA-seq libraries. This transcriptome was mapped to various genome assemblies using PASA and the numbers and lengths of valid alignments, failed alignments, and transcript assemblies were compared. In addition, the numbers and lengths of exon-only regions of the valid alignments were also extracted and compared across the assemblies.

### Gene prediction using GlimmerHMM

GlimmerHMM was used for benchmarking the assemblies. We created training sets based on *Citrus sinensis* and *C. clementina* (genes.gff3 files downloaded from http://phytozome.jgi.doe.gov/pz/portal.html), and used the inbuilt *Arabidopsis thaliana* training set to predict genes and gene structures in the neem assemblies. Both *Citrus* species were used here since they were found to be the evolutionarily closest to neem, among sequenced species (Krishnan *et al.* 2012).

### Repeat analyses

RepeatModeler v1.0.8 (Smit *et al.* 2014), employing Repeat Scout, Tandem Repeat Finder, and Recon modules, was used to construct a library of novel repeats entirely based on the neem genome. Other tools such as LTR_finder v1.0.5 (Xu and Wang 2007), TransposonPSI v08222010 (Haas 2010), and MITE-hunter v11-2011 (Han and Wessler 2010), were used to identify Long Terminal Repeats (LTRs), retrotransposons, and Miniature Inverted repeat Transposable Elements (MITEs), respectively. The neem genome assembly was masked using RepeatMasker v4.0.5 (Smit *et al.* 2014) with all these repeats and the updated plant (Viridiplantae) libraries from Repbase (Kapitonov and Jurka 2008), to estimate the nonredundant genomic repeat content. This was further classified using the RepeatClassifier module of RepeatModeler.

### Identification of FDFT1 and SQLE gene structures across assemblies

We obtained the transcript sequences corresponding to *FDFT1* and *SQLE* genes in *C. clementina* from KEGG (Kanehisa and Goto 2000; Kanehisa *et al.* 2014), and created a database of these sequences using the makeblastdb utility in the BLAST package v2.2.29 (Altschul *et al.* 1990). These genes belong to the sesqui- and tri-terpenoid biosynthesis pathways, involved in the synthesis of the commercially important compound, azadirachtin, and hence were chosen for comparative analyses here. The neem transcriptome was mapped against the database using BLAST with an Expect (E) value threshold of 0.001. The mapped neem transcripts were traced to their PASA alignments in various genome assemblies. In cases where the identified transcripts for the same reference gene aligned to multiple neem scaffolds, consensus exon−intron structures were inferred individually for each scaffold, and the one agreeing best with the *C. clementina* gene structure was considered. The gene structures for all assemblies were plotted along with the corresponding gene structure in *C. clementina* using ‘Structure Draw’ (http://www.compgen.uni-muenster.de/tools/strdraw/index.hbi?). Regions of gaps (Ns) in the assembly were highlighted in red.

All scripts used in the assembly, QC, evaluation, genome-to-transcriptome mapping, gene prediction, and repeat analyses pipeline are presented under in File S2.

### Experimental validation of FDFT1 and SQLE genes

We synthesized primers (Table S3) for *FDFT1* and *SQLE* genes to confirm whether the v2.0 assembly is indeed improved over the previous one. The primers were designed using National Center for Biotechnology Information (NCBI) Primer-BLAST (http://www.ncbi.nlm.nih.gov/tools/primer-blast/) in genic regions using v1.0 and v2.0 assemblies. We amplified the genes using the conditions (denaturation at 94° for 30 sec, followed by 35 cycles of denaturation at 94° for 30 sec, annealing at 58° for 30 sec, and extension at 68° for 8 min, followed by a final extension at 68° for 10 min). The amplified products were loaded onto a 0.8% agarose gel to visualize DNA bands.

### Data availability

The raw data described in this manuscript are submitted to the NCBI Short Read Archive under the accession numbers SRX1074131, SRX1074132, SRX1074133, and SRX1074134 (SRP013453). The authors state that all data necessary for confirming the conclusions presented in the article are represented fully within the article.

## Results

### Quality comparison across all versions of neem genome assembly

We compared the correctness and completeness of all the assembly versions based on three measures:

1. Assembly statistics using QUAST
2. Metrics from transcriptome-to-assembly alignment using PASA
3. Gene and gene structure prediction based on three different training sets using GlimmerHMM

The first measure strictly quantifies the completeness of the assembly, while the middle one mainly quantifies the correctness of the assembly, and its completeness to the extent that the draft transcriptome is complete, and the last measure quantifies the completeness of the assembly, but also its correctness, with the assumption that the genes and gene structures in the organisms used as training sets are present, as is, in the neem genome. Detailed metrics from all the benchmarking tools are provided in Table S2.

### Comparison of assembly statistics

**Figure 1:**
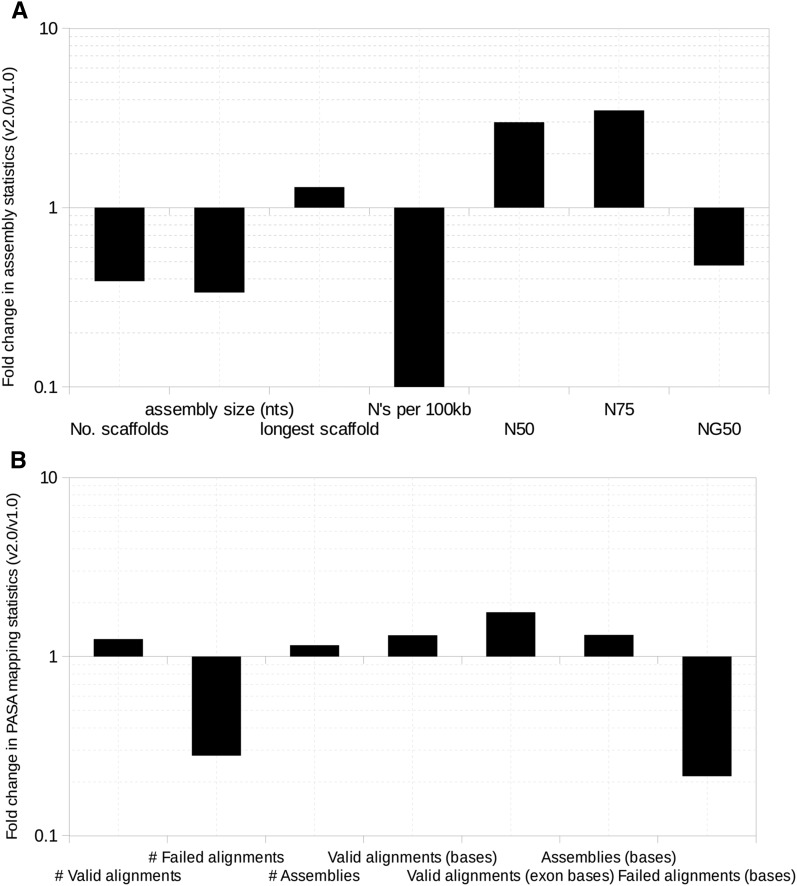
Improvements (fold change between current, v2.0, over the previous, v1.0, assembly) in various (A) assembly statistics and (B) PASA mapping statistics. The Y-axis is plotted on a logarithmic scale and the minor grids conform to uniform intervals on positive and negative Y-axis.

Overall, assembly statistics improved with Platanus over SOAPdenovo or SOAPdenovo2 (Figure 1 and Table S2), with the best assembly (v2.0) produced by Platanus using a combination of all duplicate-removed Illumina read libraries and error-corrected (kmer = 32) PacBio library in all three stages—assembly, scaffolding, and gap-closing. The scaffold numbers and the assembly size here were reduced by 2.6- and 3-fold, respectively, over those from the earlier draft assembly (v1.0; Figure 1). The assembly using uncorrected PacBio reads, in combination with Illumina libraries (P.ucPB), resulted in the longest scaffold (12,211,325 bases). However, other important quality metrics were compromised for this assembly. N50 and N75 were highest for Platanus assembly using all Illumina-only reads (P; 4,002,232 and 1,489,583 bases, respectively). The v2.0 assembly revealed a 13.4-fold reduction in gaps over the v1.0 assembly (an average of 5414.21 Ns per 100 kb, Figure 1A) and a 2.26-fold lowered NG50. Incidentally, the NG50 for the Platanus assembly using Illumina-only reads (P; 1,587,838 bases) was comparable to that using SOAPdenovo (v1.0; 1,663,167 bases). Almost 60% of each assembly was covered at 5X when PacBio reads were assembled, along with Illumina read libraries, using SOAPdenovo2 or Platanus (Table S2).

### Comparison of transcriptome-to-genome alignment metrics

The numbers and cumulative lengths of all valid alignments and PASA assemblies were highest at 77,635 and 61,292, ∼100 Mb and ∼99 Mb, respectively, for the v2.0 assembly (Table S2). The cumulative size of valid exonic alignments was also highest at ∼48 Mb for this assembly, and the corresponding numbers and lengths of all failed alignments were least at 6584 and ∼32 Mb, respectively (Table S2). The overall valid alignments increased 1.25-fold, and the ones in exons increased by 1.95-fold for the updated (v2.0) assembly over the old one (v1.0) (Figure 1B). Failed alignments went down by 3.5- and 5.9-fold in number and cumulative size, respectively (Figure 1B).

### Comparison of predicted genes

**Figure 2:**
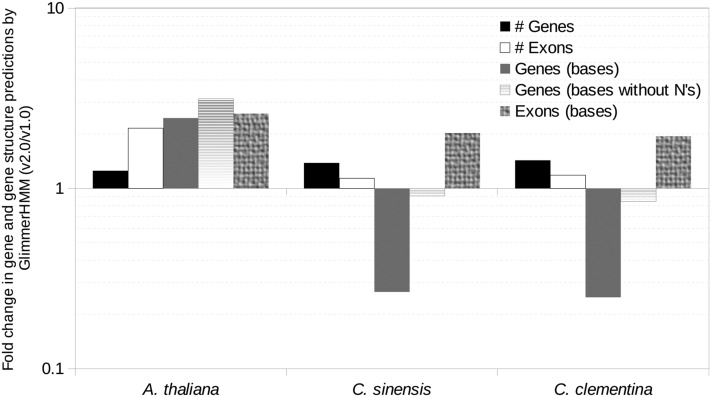
Improvements (fold change between current, v2.0, over the previous, v1.0, assembly) in the numbers (#s) and sizes (bases) of gene and exon predictions from GlimmerHMM. The Y-axis is plotted on a logarithmic scale and the minor grids conform to uniform intervals on positive and negative Y-axis.

We found the highest number of predicted genes and exons using training sets from any of the three organisms (*A. thaliana*, *C. sinensis*, *C. clementina*), with the v2.0 assembly (Table S2 and Figure 2). The cumulative length of all predicted genes was highest for this assembly (68,723,917 bases) when *A. thaliana* was used as the training set. When *Citrus* species were used as training sets, however, the v1.0 assembly resulted in the highest cumulative predicted gene lengths (473,787,912 and 431,305,649 bases, respectively, with *C. sinensis* and *C. clementina*). The predicted gene lengths were comparable between both the assemblies after excluding gaps, suggesting this to be mostly a result of misassembly (Figure 2).

**Figure 3:**
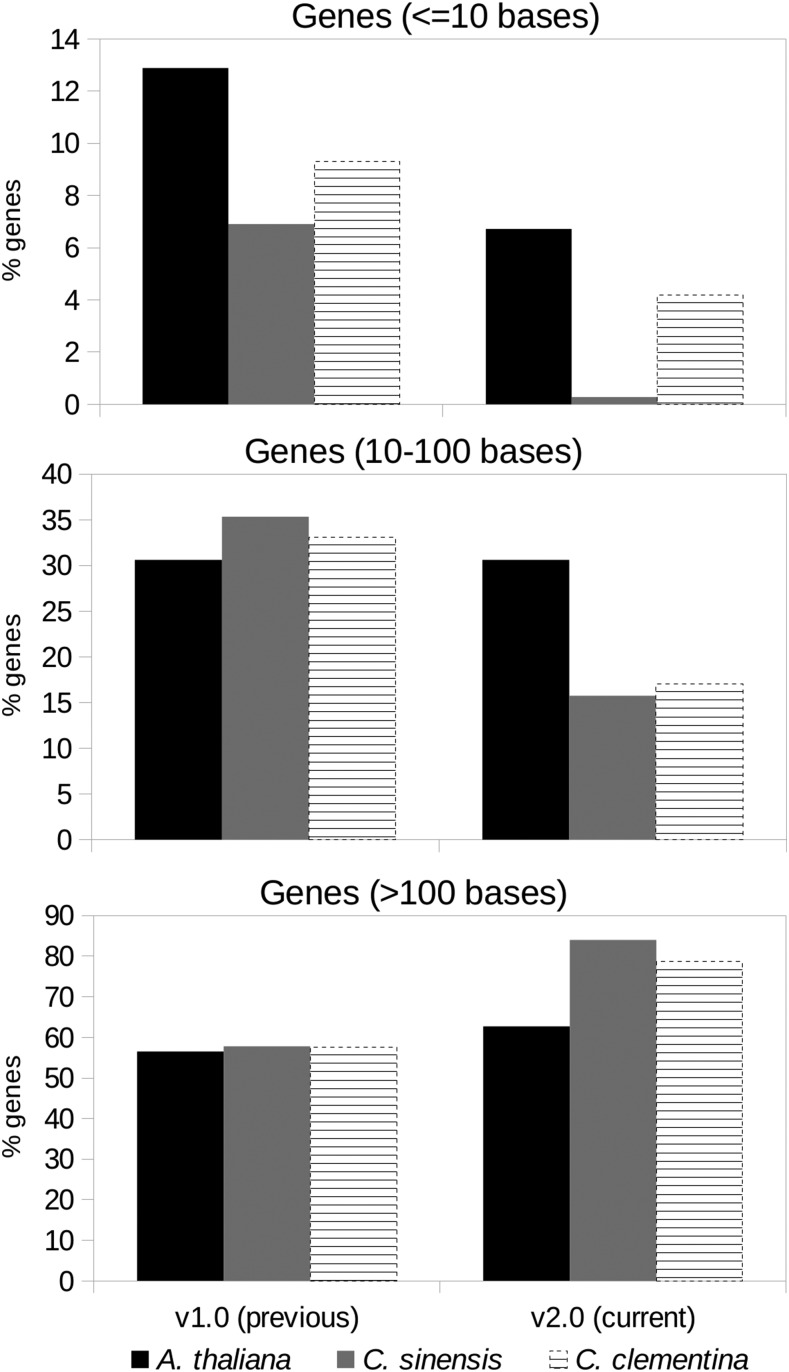
Proportion (%) of gene-bearing scaffolds/contigs with gene predictions of lengths <10 bases, 10−100 bases, and >100 bases, for *A. thaliana*, *C. sinensis*, and *C. clementina* training sets.

We found an abundance of smaller (<100 bases) mRNAs and exons in gene predictions in the v1.0 assembly, especially with *Citrus* training sets, which were substantially reduced in the v2.0 assembly (Figure 3). In contrast, the longer mRNAs were more abundant in the latter assembly, with *Citrus* training sets, an indication of improvement in the assembly.

### Comparison of gene structures of FDFT1 and SQLE across various assemblies

**Figure 4:**
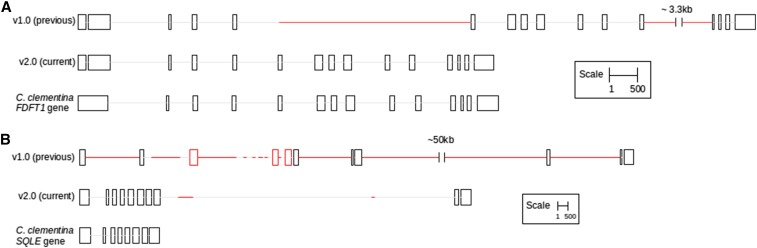
Comparison of v1.0 and v2.0 assemblies for (A) *FDFT1* and (B) *SQLE* genes. The *FDFT1* and *SQLE* transcripts from *C. clementina* were mapped to the representative Trinity-assembled *A. indica* transcriptome using NCBI BLAST (E-value 0.001). The transcripts were traced to their neem genomic scaffold mappings from PASA, in order to extract the exon−intron structures of the corresponding genes. In the figure, boxes and lines denote exons and introns, respectively, and the red regions denote gaps in the assemblies. The scales are different for *FDFT1* and *SQLE* and are, therefore, indicated individually.

In order to demonstrate the biological significance of the improved assembly, we used *FDFT1* and *SQLE* genes, two important genes involved in the sesqui- and triterpenoid biosynthesis pathways. We observed that the gene structures of *FDFT1* and *SQLE* were more complete and accurate in the improved v2.0 assembly when compared to the v1.0 assembly (Figure 4 and Figure S2). Using Platanus alone, and augmenting the libraries with additional short Illumina mate-pair libraries yielded a better *FDFT1* gene structure. Similarly, using Platanus as an assembler along with pre-and postprocessing yielded a better assembly of the multi-isoform *SQLE* gene.

We found that the read support offered by Illumina and PacBio libraries for *FDFT1* and *SQLE* genes to be stronger and more contiguous in the case of the v2.0 assembly as compared to the v1.0 (Figure S3A and 3B). Additionally, we used Integrative Genomics Viewer (IGV) to visualize the mapped reads to earlier and current scaffolds containing these two genes (Figure S3) to demonstrate the superiority of the v2.0 assembly over the earlier version (v1.0). As shown in Figure S3, additional short and long reads along with the usage of the assembler, Platanus, resulted in gene assemblies that are more contiguous (gray boxes) and with lesser gaps (white boxes) than the earlier (v1.0) assembly. We further experimentally verified both versions of the assemblies by designing primers to amplify two key genes, *FDFT1* and *SQLE*. We expected to obtain amplified products with sizes of 4 kb, 7.3 kb, and 3.8 kb (for partial *FDFT1*, full *FDFT1*, and partial *SQLE* genes, respectively) as per our v2.0 assembly (Table S4). Had the earlier version of the assembly (v1.0) been correct, we were expecting to obtain much higher sizes of the bands (11 kb or higher) for both *FDFT1* and *SQLE* genes (Figure S4A and Table S4). As shown in Figure S4B, it is clear that the v2.0 assembly is indeed an improved and correct one for these two genes over the previous assembly.

### Estimation of repeat content

The repeat content was estimated to be 54,375,206 bases (24.15% of v2.0), which is higher than the 47,427,034 bases reported earlier (13.03% of v1.0). We further classified the repeats into distinct classes, as shown in Table S5.

## Discussion

Here, we report an improved genome assembly of *A. indica* and provide quantitative evidence on various parameters in support of the improved assembly. The current assembly benefits from using additional Illumina mate-pair reads and long reads from PacBio, a third generation sequencing platform. Additionally, we have used Platanus, a tool designed to assemble heterozygous genomes, such as that of neem (Figure S1), better, and an algorithm that uses short reads to correct the errors in long reads. Finally, we have used updated and near complete training sets from closely related plant species to predict gene structures, and an equally enriched and updated repeat library to predict repeat sequences in the neem genome.

In our study, we employed PASA and GlimmerHMM to benchmark the assemblies, both of which have their limitations in the current context. PASA assumes that the transcriptome is free of misassembly errors. The caveat with GlimmerHMM, is that the gaps and errors in the genomic assembly extends to the predictions (Figure 2). We found that the number of gene predictions decreased across assemblies, postredundancy removal using cd-HIT-EST (Li and Godzik 2006). Additionally, the gene predictions are only as good as the training sets used. Presence of a large number of very short, possibly spurious, exons in the *C. clementina* training set manifested in a large number of similar predictions in the neem assembly (Figure 3). However, as expected, either these did not align to the neem transcriptome, or a large fraction of those that aligned did not meet the validity criteria set by PASA, suggesting incorrect predictions. This implied a larger number of gene predictions not to be an indicator of correct or complete assembly in neem. Instead, integration of results from multiple tools, preferably using additional information from orthogonal high-throughput platforms such as RNA-seq, and experimental validation offered better benchmarking.

The presence of duplicate reads may give false assurance to the assembler in terms of artificially inflated read depth. Hence, removing exact read duplicates reduced the number of misassemblies. Interestingly, we found that the assembly with SOAPdenovo2, after duplicate removal (S2), displayed worse statistics, but much improved transcriptome-genome mappings using PASA (Table S2). SOAPdenovo, using fewer Illumina libraries, and without a duplicate removal step (v1.0), also displayed suboptimal assembly statistics but a good NG50 number (Figure 1). This, most likely, is due to an abundance of gaps in the assembly, inflating the assembly size. Incidentally, the NG50 numbers for assemblies using libraries from the same platform were comparable (Table S2). Such observations caution against deriving conclusions regarding best assembly based solely on assembly statistics tools, such as QUAST. Exploring the finer details of individual genomic features, instead of macro-level statistics like NG50, may provide a better estimate of the improvement in the assembly quality, as exemplified by the improved assembly of two enzymes, *FDFT1* and *SQLE* catalyzing key stages of the biosynthetic route to sterols and triterpenes (Thimmappa *et al.*, 2014), in the improved neem assembly (Figure S3 and Figure S4). Relying solely on sequence similarity-based approaches for gene identification can result in incomplete and/or inaccurate structural annotations. Using BLAST against *C. clementina* transcripts, with a stringent E-value threshold of 0.001, identified only portions of the *FDFT1* and *SQLE* genes in our scaffolds, making us falsely deduce that we had assembled only certain exons from these genes. This would be particularly true for structurally conserved genes, which have few very important, and, therefore, conserved domains. In such genes, variable domains might not have significant sequence homology to the reference database(s) that include sequences from other species, causing the genes to not be annotated in their entirety. Therefore, our approach of using the sequence similarity between *C. clementina* and neem transcripts to trace back the entire gene sequence structure and combining both reference- and *de novo*-based identification techniques is a better one (Figure 4).

In conclusion, genome assemblies need to be updated continuously by implementing accurate computational algorithms and supplementing with experimental evidence to obtain error-free and near complete assemblies. The process of obtaining accurate genome assembly is a dynamic and continuous process that needs to be undertaken, in our opinion, by groups or communities that have produced the first draft sequence of various genomes. This will facilitate research in genomics and create public resources to understand gene structure and function in plants better.

## Supplementary Material

Supplemental Material
