# Corrigendum

**DOI:** 10.1534/g3.119.400327

**Published:** 2019-06-26

In the article by N. M. Krishnan, P. Jain, S. Gupta, A. K. Hariharan, and B. Panda (*G3: Genes|Genomes|Genetics* 6(7): 1835-1840) entitled “An Improved Genome Assembly of *Azadirachta indica* A. Juss.,” on pages 1837-1838, the fasta assembly for the analysis was not made available as part of the Data Availability Statement, which has now been corrected to include the following sentence: The assembly is deposited in figshare: https://figshare.com/articles/AZ_fa/8080733.

