# Supplementary material for: An Improved Genome Assembly of Azadirachta indica A. Juss
Source: G3 (Bethesda). 2016 Apr 18;6(7):1835–40. doi: 10.1534/g3.116.030056 (PMC4938638; doi:10.1534/g3.116.030056)
Supplement: Supplemental Material [file supp_6_7_1835__index.html]

An Improved Genome Assembly of Azadirachta indica A. Juss. — Supplemental Material 

# An Improved Genome Assembly of *Azadirachta indica* A. Juss.

## Supplemental Material for Krishnan *et al.*, 2016

**Files in this Data Supplement:**

- File S1 - NUCMER based mapping of smaller Illumina reads coming from a single long PacBio read, to the assembly. (.pdf, 58 KB)
- File S2 - Supplementary Scripts. (.pdf, 60 KB)
- Figure S3 - IGV snapshots of the alignments of various read libraries to the *SQLE* (A) and *FDFT1* (B) genes discovered in the earlier version (v1.0), current version (v2.0) and intermediate assemblies (S2 and P), performed by using SOAPdenovo2 (S2) and Platanus (P), respectively. Read alignment was performed using Novoalign v3.03 and the assembled sequences are represented (grey area: properly assembled regions, white boxes: gaps)and the 'gaps' are denoted by blanks, as exemplified by the arrows. (.pdf, 1,917 KB)
- Figure S1 - kmer frequency curve. The frequency (%) of 17-mers is plotted as a function of the number of times they occur across paired-end libraries. The peaks for heterozygous, homozygous and repetitive kmers are highlighted by arrows. (.tif, 145 KB)
- Figure S2 - Comparison across assemblies for A: *FDFT1* and B: *SQLE* genes. The *FDFT1* and *SQLE* transcripts from *C. clementina* were mapped to the representative Trinity- assembled *A. indica* transcriptome using NCBI BLAST (E-value 0.001). The transcripts were traced to their neem genomic scaffold mappings from PASA, in order to extract the exon-intron structures of the corresponding genes. In the figures, boxes and lines denote exons and introns, respectively, and the red regions denote gaps in the assemblies. The scales are different for *FDFT1* and *SQLE* and are, therefore, indicated individually. (.tif, 2,125 KB)
- Figure S4 - Experimental validation for *FDFT1* and *SQLE* gene assemblies. A. Cartoon with expected sizes of the bands for both genes, as per earlier version (v1.0) or the current version (v2.0) of the assembly. As it is shown by amplification of both partial and full-length *FDFT1* and partial *SQLE* genes, the current version (v2.0) is the right assembly for the genes. (.tif, 699 KB)
- Table S1 - Details of sequencing libraries. PE: short-insert paired end, MP: long-insert mate pair libraries. (.xls, 22 KB)
- Table S2 - Performance comparison using QUAST, PASA and GlimmerHMM across various assemblies. (.xls, 67 KB)
- Table S3 - Read mapping statistics to v2.0 and v1.0 assemblies, by Novoalign v3.04 (http://www.novocraft.com/). (.xls, 21 KB)
- Table S4 - Primers designed for PCR to amplify partial and complete *SQLE* and *FDFT1* genes. (.xls, 21 KB)
- Table S5 - Repeat element classification. (.xls 22 KB)
