# Supplementary material for: An Improved Genome Assembly of Azadirachta indica A. Juss
Source: G3 (Bethesda). 2016 Apr 18;6(7):1835–40. doi: 10.1534/g3.116.030056 (PMC4938638; doi:10.1534/g3.116.030056)
Supplement: Supplemental Material [file supp_g3.116.030056_FileS1.pdf]

File S1. NUCMER based mapping of smaller Illumina reads coming from a single long PacBio read, to the assembly.

NUCMER (Read 1)

[S1]	[E1]	[S2]	[E2]	[LEN1]	[LEN2]	[% IDY]	[TAGS]	
22	100	726	648	79	79	100.00	m121022_124828_42181_c100388272550000001523034010251242_s1_p0/7/0_806_190_681_12_1_0	scaffold24417_len726_cov2219_single
1	100	712	613	100	100	100.00	m121022_124828_42181_c100388272550000001523034010251242_s1_p0/7/0_806_225_666_14_1_0	scaffold24417_len726_cov2219_single
1	100	693	594	100	100	100.00	m121022_124828_42181_c100388272550000001523034010251242_s1_p0/7/0_806_244_671_22_1_0	scaffold24417_len726_cov2219_single
10	87	78	1	78	78	100.00	m121022_124828_42181_c100388272550000001523034010251242_s1_p0/7/0_806_55_488_4_1_0	scaffold23831_len619_cov1962_single

NUCMER (Read 2)

[S1]	[E1]	[S2]	[E2]	[LEN1]	[LEN2]	[% IDY]	[TAGS]	
1	100	306	405	100	100	100.00	m121022_124828_42181_c100388272550000001523034010251242_s1_p0/7/0_806_106_532_11_1_1	scaffold24417_len726_cov2219_single
1	100	276	375	100	100	100.00	m121022_124828_42181_c100388272550000001523034010251242_s1_p0/7/0_806_111_562_6_1_1	scaffold24417_len726_cov2219_single
1	100	260	359	100	100	100.00	m121022_124828_42181_c100388272550000001523034010251242_s1_p0/7/0_806_134_578_2_1_1	scaffold24417_len726_cov2219_single
1	100	235	334	100	100	100.00	m121022_124828_42181_c100388272550000001523034010251242_s1_p0/7/0_806_140_603_15_1_1	scaffold24417_len726_cov2219_single
1	100	232	331	100	100	100.00	m121022_124828_42181_c100388272550000001523034010251242_s1_p0/7/0_806_146_606_19_1_1	scaffold24417_len726_cov2219_single
1	100	157	256	100	100	100.00	m121022_124828_42181_c100388272550000001523034010251242_s1_p0/7/0_806_190_681_12_1_1	scaffold24417_len726_cov2219_single
1	10	17	27	100	100	100.	m121022_124828_42181_c100388272550000001523034010251242_s1_p0/7	scaffold24417_len726_cov22

	0	2	1			00	/0_806_225_666_14_1_1	19_single
1	10<br>0	34<br>9	44<br>8	100	100	100.<br>00	m121022_124828_42181_c100388272550000001523034010251242_s1_p0/7<br>/0_806_22_489_25_1_1	scaffold24417_len726_cov22<br>19_single
1	10<br>0	16<br>7	26<br>6	100	100	100.<br>00	m121022_124828_42181_c100388272550000001523034010251242_s1_p0/7<br>/0_806_244_671_22_1_1	scaffold24417_len726_cov22<br>19_single
1	10<br>0	35<br>6	45<br>5	100	100	100.<br>00	m121022_124828_42181_c100388272550000001523034010251242_s1_p0/7<br>/0_806_2_482_17_1_1	scaffold24417_len726_cov22<br>19_single
1	10<br>0	35<br>0	44<br>9	100	100	100.<br>00	m121022_124828_42181_c100388272550000001523034010251242_s1_p0/7<br>/0_806_55_488_4_1_1	scaffold24417_len726_cov22<br>19_single
1	10<br>0	31<br>1	41<br>0	100	100	100.<br>00	m121022_124828_42181_c100388272550000001523034010251242_s1_p0/7<br>/0_806_65_527_8_1_1	scaffold24417_len726_cov22<br>19_single
