# Supplementary material for: An Improved Genome Assembly of Azadirachta indica A. Juss
Source: G3 (Bethesda). 2016 Apr 18;6(7):1835–40. doi: 10.1534/g3.116.030056 (PMC4938638; doi:10.1534/g3.116.030056)
Supplement: Supplemental Material [file supp_g3.116.030056_FileS2.pdf]

File S2. Supplementary Scripts

1. Assemble using SOAPdenovo 2.0

/Apps/SOAPdenovo2/SOAPdenovo2-src-r223/SOAPdenovo-63mer all -s config -K 31 -d 9 -F -R -o 64 -p 12

```
config:
[LIB]
avg_ins=350
reverse_seq=0
asm_flags=3
rd_len_cutoff=76
rank=1
pair_num_cutoff=3
map_len=32
q1=/storage/fastqs/s_1234_1.fastq.normalized_K25_C50_pctSD200.fq
q2=/storage/fastqs/s_1234_2.fastq.normalized_K25_C50_pctSD200.fq

[LIB]
avg_ins=350
reverse_seq=0
asm_flags=3
rd_len_cutoff=76
rank=2
pair_num_cutoff=3
map_len=32
q1=/storage/fastqs/s_567_1.fastq.normalized_K25_C50_pctSD200.fq
q2=/storage/fastqs/s_567_2.fastq.normalized_K25_C50_pctSD200.fq

[LIB]
avg_ins=1500
reverse_seq=1
asm_flags=3
rd_len_cutoff=36
rank=3
pair_num_cutoff=3
```

```
map_len=32
q1=/storage/fastqs/s34_1.fastq.normalized_K25_C50_pctSD200.fq
q2=/storage/fastqs/s34_2.fastq.normalized_K25_C50_pctSD200.fq

[LIB]
avg_ins=3000
reverse_seq=1
asm_flags=3
rd_len_cutoff=36
rank=4
pair_num_cutoff=3
map_len=32
q1=/storage/fastqs/s56_1.fastq.normalized_K25_C50_pctSD200.fq
q2=/storage/fastqs/s56_2.fastq.normalized_K25_C50_pctSD200.fq

[LIB]
avg_ins=4000
reverse_seq=1
asm_flags=3
rd_len_cutoff=100
rank=5
pair_num_cutoff=3
map_len=32
q1=/storage/fastqs/Az_3.5_4.5KB_R1.fastq.normalized_K25_C50_pctSD200.fq
q2=/storage/fastqs/Az_3.5_4.5KB_R2.fastq.normalized_K25_C50_pctSD200.fq

[LIB]
avg_ins=6000
reverse_seq=1
asm_flags=3
rd_len_cutoff=100
rank=6
pair_num_cutoff=3
map_len=32
q1=/storage/fastqs/Az_5_7KB_R1.fastq.normalized_K25_C50_pctSD200.fq
q2=/storage/fastqs/Az_5_7KB_R2.fastq.normalized_K25_C50_pctSD200.fq

[LIB]
avg_ins=10000
reverse_seq=1
asm_flags=3
rd_len_cutoff=100
rank=7
pair_num_cutoff=3
map_len=32
q1=/storage/fastqs/Az_8_11KB_R1.fastq.normalized_K25_C50_pctSD200.fq
q2=/storage/fastqs/Az_8_11KB_R2.fastq.normalized_K25_C50_pctSD200.fq

[LIB]
avg_ins=10000
reverse_seq=1
asm_flags=3
```

rd_len_cutoff=36
rank=8
pair_num_cutoff=3
map_len=32
q1=/storage/fastqs/10kb_R1.fastq_30012014_QC_passed.fastq.normalized_K25_C50_pctSD200.fq
q2=/storage/fastqs/10kb_R2.fastq_30012014_QC_passed.fastq.normalized_K25_C50_pctSD200.fq

[LIB]
avg_ins=350
reverse_seq=0
asm_flags=3
rd_len_cutoff=76
rank=9
pair_num_cutoff=3
map_len=32
q1=/storage/fastqs/hybrid.fasta_1_350_35_5.0_100.fq
q2=/storage/fastqs/hybrid.fasta_2_350_35_5.0_100.fq

2. Assemble using Platanus:

assemble:

/Apps/platanus assemble -o P.rmDUP.ecPB.32 -f
/storage/fastqs/s_1234_1.fastq.normalized_K25_C50_pctSD200.fq
/storage/fastqs/s_1234_2.fastq.normalized_K25_C50_pctSD200.fq
/storage/fastqs/s_567_1.fastq.normalized_K25_C50_pctSD200.fq
/storage/fastqs/s_567_2.fastq.normalized_K25_C50_pctSD200.fq
/storage/fastqs/hybrid_1_350_35_5.0_100.fq /storage/fastqs/hybrid_2_350_35_5.0_100.fq -k 32 -s
5 -t 12 -m 100

scaffold:

/Apps/platanus scaffold -o P.rmDUP.ecPB.32 -c P.rmDUP.ecPB.32_contig.fa -b
P.rmDUP.ecPB.32_contigBubble.fa -IP1
/storage/fastqs/s_1234_1.fastq.normalized_K25_C50_pctSD200.fq
/storage/fastqs/s_1234_2.fastq.normalized_K25_C50_pctSD200.fq -IP2
/storage/fastqs/s_567_1.fastq.normalized_K25_C50_pctSD200.fq
/storage/fastqs/s_567_2.fastq.normalized_K25_C50_pctSD200.fq -IP3
/storage/fastqs/hybrid_1_350_35_5.0_100.fq /storage/fastqs/hybrid_2_350_35_5.0_100.fq -OP4
/storage/fastqs/s34_1.fastq.normalized_K25_C50_pctSD200.fq
/storage/fastqs/s34_2.fastq.normalized_K25_C50_pctSD200.fq -OP5
/storage/fastqs/s56_1.fastq.normalized_K25_C50_pctSD200.fq
/storage/fastqs/s56_2.fastq.normalized_K25_C50_pctSD200.fq -OP6
/storage/fastqs/10kb_R1.fastq_30012014_QC_passed.fastq.normalized_K25_C50_pctSD200.fq
/storage/fastqs/10kb_R2.fastq_30012014_QC_passed.fastq.normalized_K25_C50_pctSD200.fq -
OP7 /storage/fastqs/Az_3.5_4.5KB_R1.fastq.normalized_K25_C50_pctSD200.fq
/storage/fastqs/Az_3.5_4.5KB_R2.fastq.normalized_K25_C50_pctSD200.fq -OP8
/storage/fastqs/Az_5_7KB_R1.fastq.normalized_K25_C50_pctSD200.fq
/storage/fastqs/Az_5_7KB_R2.fastq.normalized_K25_C50_pctSD200.fq -OP9
/storage/fastqs/Az_8_11KB_R1.fastq.normalized_K25_C50_pctSD200.fq
/storage/fastqs/Az_8_11KB_R2.fastq.normalized_K25_C50_pctSD200.fq -n1 315 -n2 315 -n3 315
-n4 1350 -n5 2700 -n6 9000 -n7 3500 -n8 5000 -n9 8000 -a1 350 -a2 350 -a3 350 -a4 1500 -a5 3500

```
-a6 10000 -a7 4000 -a8 6000 -a9 9500 -d1 35 -d2 35 -d3 35 -d4 150 -d5 350 -d6 1000 -d7 400 -d8 600 -d9 950
```

3. SINC to generate Illumina-like 100bp reads with 350±35 bp insert size from PacBio reads

```
/Apps/SInC/SInC_readGen -D 350 -S 35 -C 5 -T 10 -R 100 hybrid.fasta
/Apps/SInC/100_bp_read_1_profile.txt /Apps/SInC/100_bp_read_2_profile.txt 1> sinc.log 2>&1
```

4. LoRDEC to error-correct PacBio reads using Illumina libraries

```
/Apps/LoRDEC-0.4.1/lordec-correct -T 4 -i /storage/fastqs/filtered_subreads.fastq -2
s_1234_1.fastq s_1234_2.fastq s_567_1.fastq s_567_2.fastq -k 19 -o hybrid -s 3
```

5. Assembly QC using QUAST

```
python quast.py -t 4 --scaffolds P.rmDUP.ecPB.32.fa
```

6. Mapping genome to transcriptome using PASA

```
sed 's/ path=\[.*\]$//g' Trinity.fasta | sed 's/[ =]/_/g' > Trinity_headerMod.fasta
/Apps/PASA_r20140417/seqclean/seqclean/seqclean Trinity_headerMod.fasta
/Apps/PASA_r20140417/scripts/Launch_PASA_pipeline.pl -c alignAssembly.config -C -R -g
P.rmDUP.ecPB.32.fa-t Trinity_headerMod.fasta.clean -T -u Trinity_headerMod.fasta --ALIGNERS
gmap --CPU 12 1>pasa.out 2>pasa.err &
```

7. Training set creation and Gene prediction using GlimmerHMM-Train and GlimmerHMM

training with *C. sinsensis*

```
# formatting exon file for input to trainGlimmerHMM
egrep "exon|mRNA" Csinensis_154_gene_exons.gff3 |cut -f 1,3,4,5,7 | sed 's/.*\tmRNA\t.*//'|awk -
F"\t" '{if($5=="-") {print $1"\t"$4"\t"$3;} else {print $1"\t"$3"\t"$4}}' | sed 1d >
Csinensis_154_gene_exons_forGlimmerHMM.tsv
/Apps/GlimmerHMM/GlimmerHMM3.0.4/train/trainGlimmerHMM Csinensis_154.fa
Csinensis_154_gene_exons_forGlimmerHMM.tsv -d Csinensis.glimmerTraining
```

training with *C. clementina*

```
# formatting exon file for input to trainGlimmerHMM
egrep "exon|mRNA" Cclementina_182_v1.0.gene_exons.gff3 |cut -f 1,3,4,5,7 | sed
's/.*\tmRNA\t.*//'|awk -F"\t" '{if($5=="-") {print $1"\t"$4"\t"$3;} else {print $1"\t"$3"\t"$4}}' | sed
1d > Cclementina_182_v1.0.gene_exons_forGlimmerHMM.tsv
/Apps/GlimmerHMM/GlimmerHMM3.0.4/train/trainGlimmerHMM Cclementina_182_v1.fa
Cclementina_182_v1.0.gene_exons_forGlimmerHMM.tsv -d Cclementina.glimmerTraining
```

```
# running GlimmerHMM with Arabidopsis
/Apps/GlimmerHMM/GlimmerHMM3.0.4_mod/bin/glimmhmm.pl
/Apps/GlimmerHMM/GlimmerHMM3.0.4_mod/bin/glimmerhmm_linux_x86_64
P.rmDUP.ecPB.32.fa /Apps/GlimmerHMM/GlimmerHMM3.0.4_mod/trained_dir/arabidopsis -g >
```

```
P.rmDUP.ecPB.32.arabidopsis.glimmerhmm.txt 2>glimmer.arabidopsis.err
# running GlimmerHMM with C. sinensis
/Apps/GlimmerHMM/GlimmerHMM3.0.4_mod/bin/glimmhmm.pl
/Apps/GlimmerHMM/GlimmerHMM3.0.4_mod/bin/glimmerhmm_linux_x86_64
P.rmDUP.ecPB.32.fa Csinensis.glimmerTraining -g >
P.rmDUP.ecPB.32.csinensis.glimmerhmm.txt 2>glimmer.csinensis.err

# running GlimmerHMM with C. clementina
/Apps/GlimmerHMM/GlimmerHMM3.0.4_mod/bin/glimmhmm.pl
/Apps/GlimmerHMM/GlimmerHMM3.0.4_mod/bin/glimmerhmm_linux_x86_64
P.rmDUP.ecPB.32.fa Cclementina.glimmerTraining -g >
P.rmDUP.ecPB.32.cclementina.glimmerhmm.txt 2>glimmer.cclementina.err
```

8. Repeat analyses pipeline

```
# Mite-hunter

perl /Apps/MITE_Hunter/MITE_Hunter_manager.pl -i P.rmDUP.ecPB.32.fa -g AZ -S 12345678 -c
12

# transposon-PSI
nohup /Apps/TransposonPSI_08222010/transposonPSI.pl P.rmDUP.ecPB.32.fa nuc

# LTR_finder
nohup /Apps/ltrFinder_1.0.5/ltr_finder -w 0 P.rmDUP.ecPB.32.fa >
P.rmDUP.ecPB.32.ltrFinder1.log 2> P.rmDUP.ecPB.32.err

egrep "^\[|^[35]'-LTR|^TSR" P.rmDUP.ecPB.32.ltrFinder.log | sed 's/ Len:.*//;s/.*: //;s/^\[[0-9][0-
9]*\] //;s/ - .* , .* - \([0-9][0-9]*\) \[.*/\t\1/;s/ - /\t/' | awk '{if(FNR%4==1) {scaf=$1} else
if(FNR%4==2) {start1=$1-1;end1=$2} else if(FNR%4==3) {start2=$1-1;end2=$2} else
{if($0!~/NOT FOUND/) {start1=$1-1; end2=$2;} print
scaf"\t"start1"\t"end1"\n"scaf"\t"start2"\t"end2}}' > P.rmDUP.ecPB.32.ltrFinder.bed
fastaFromBed -fi P.rmDUP.ecPB.32.fa -bed P.rmDUP.ecPB.32.ltrFinder.bed -fo
P.rmDUP.ecPB.32.ltrOut.fa

# RepeatModeler
perl /Apps/RepeatModeler/BuildDatabase -name P.rmDUP.ecPB.32 P.rmDUP.ecPB.32.faperl
/Apps/RepeatModeler/RepeatModeler -engine ncbi -database P.rmDUP.ecPB.32 1>> run.log 2>&1

# RepeatMasker
nohup /Apps/RepeatMasker/RepeatMasker -s -nolow -gff -no_is -norna -pa 16 -lib allRepeats.fa
P.rmDUP.ecPB.32.fa 1>rm.P.rmDUP.ecPB.32.log 2>&1

# RepeatClassifier
/Apps/RepeatModeler/RepeatClassifier -consensi allRepeats.fa
```
