# Supplementary figures and images for: An Improved Genome Assembly of Azadirachta indica A. Juss
Source: G3 (Bethesda). 2016 Apr 18;6(7):1835–40. doi: 10.1534/g3.116.030056 (PMC4938638; doi:10.1534/g3.116.030056)

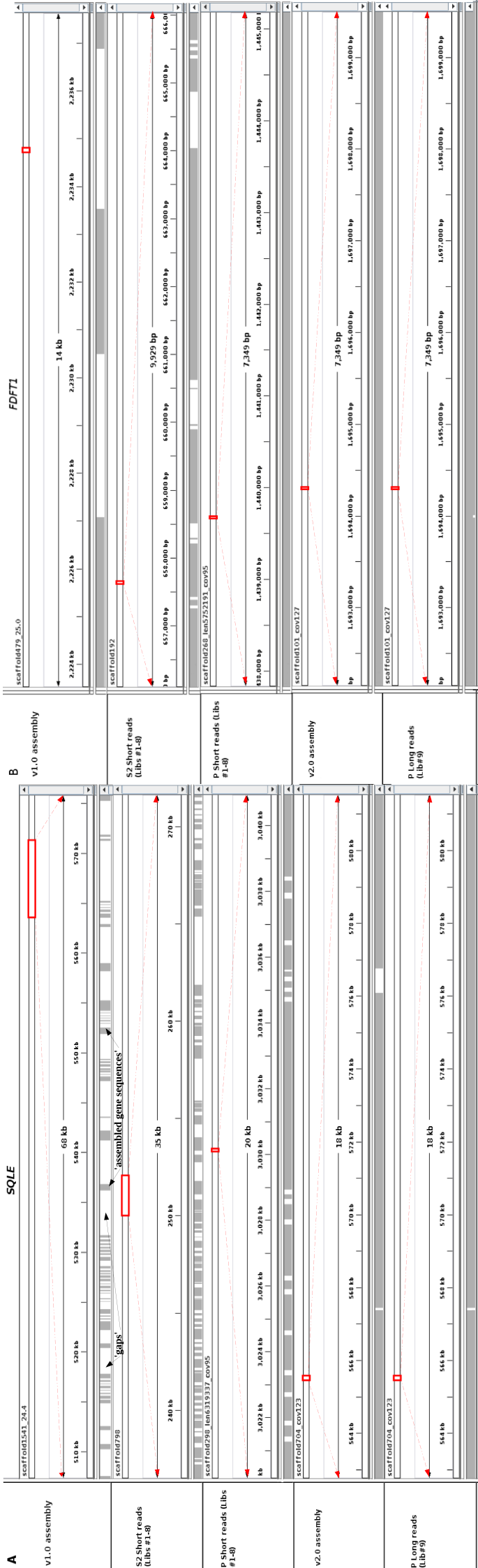

Supplementary Figure 3

Supplement: Supplemental Material [file supp_g3.116.030056_FigureS3.pdf]

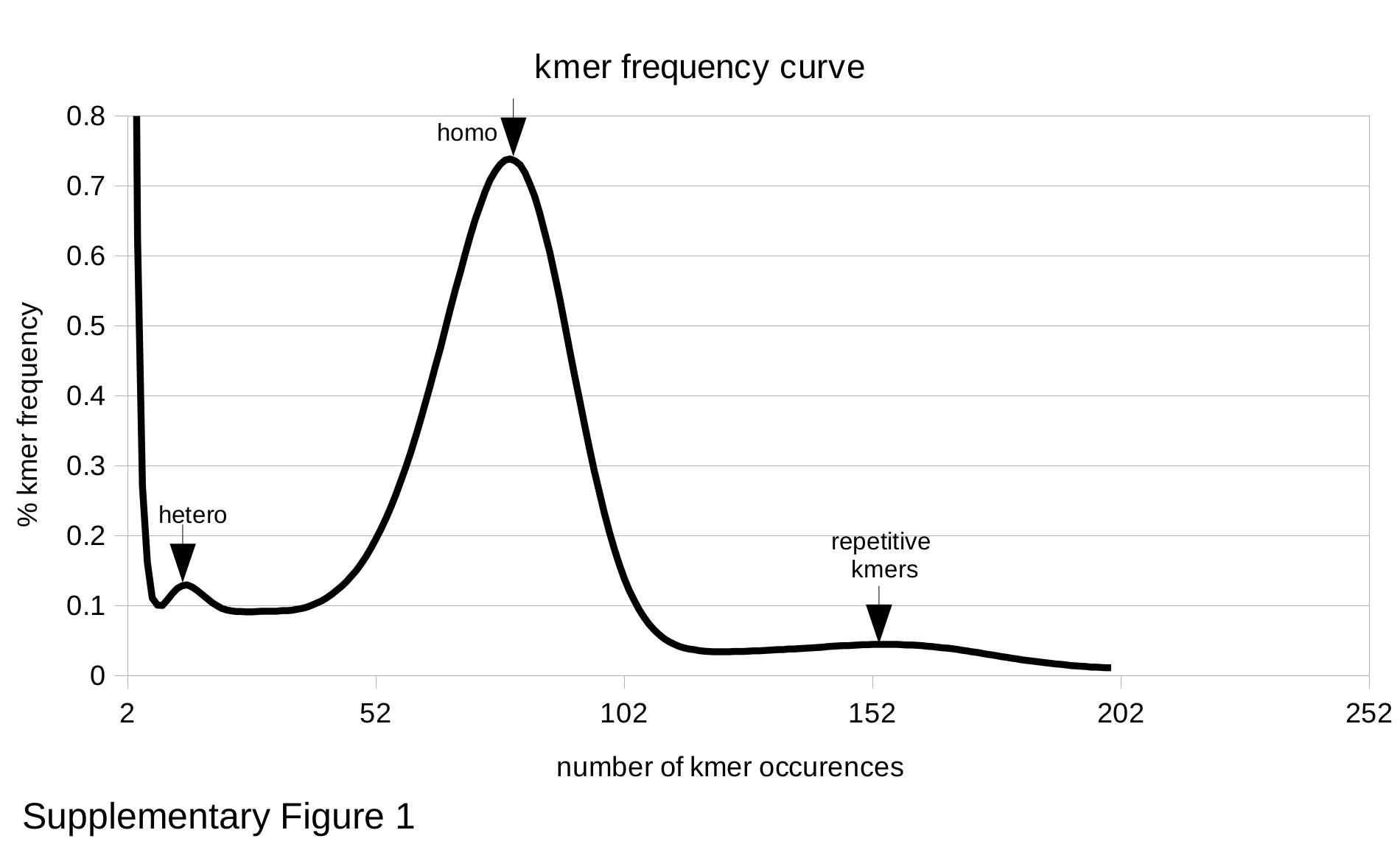

Supplement: Supplemental Material [file supp_g3.116.030056_FigureS1.tif]

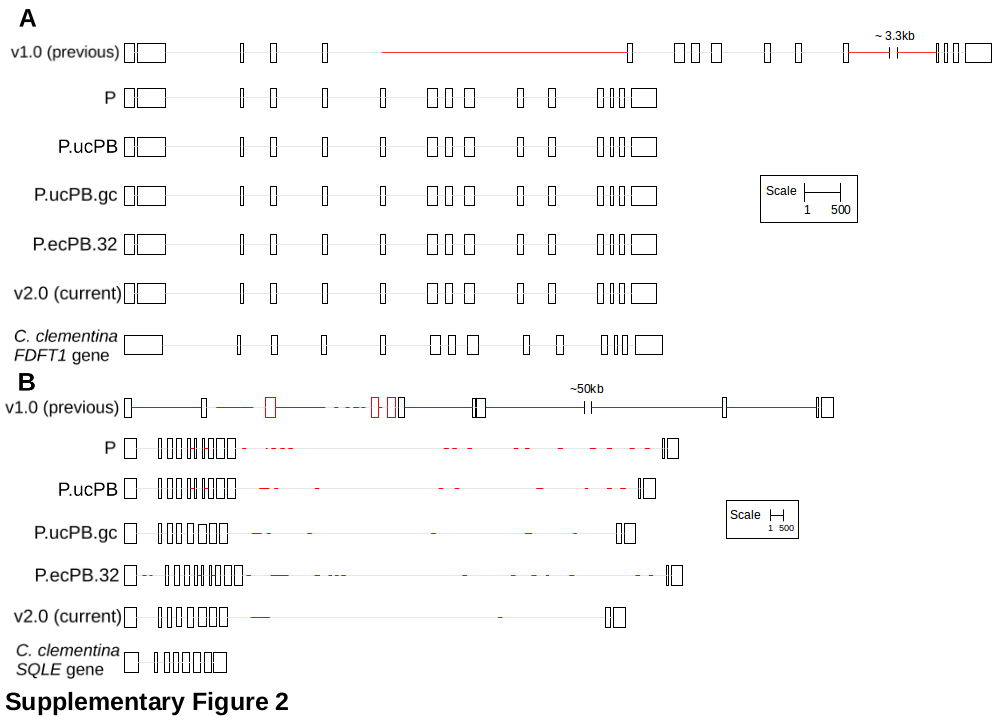

Supplement: Supplemental Material [file supp_g3.116.030056_FigureS2.tif]

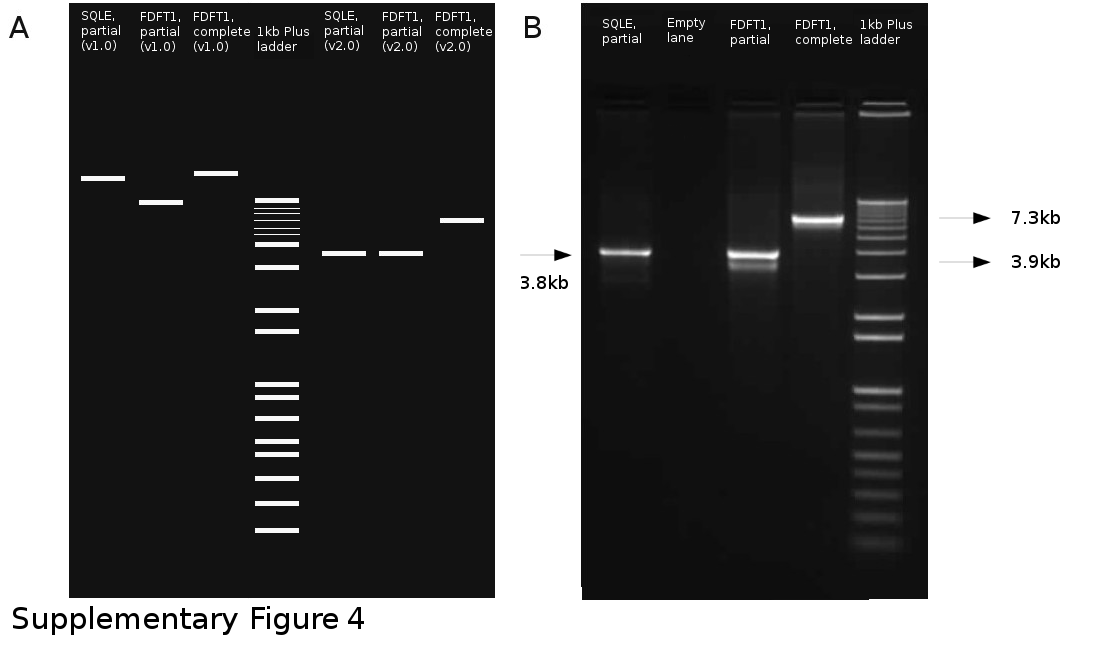

Supplement: Supplemental Material [file supp_g3.116.030056_FigureS4.tif]
